# A systematic review of factors affecting children’s right to health in cluster randomized trials in Kenya

**DOI:** 10.1186/1745-6215-15-287

**Published:** 2014-07-16

**Authors:** Elizabeth Oduwo, Sarah JL Edwards

**Affiliations:** 1 University College London, London, United Kingdom

**Keywords:** Cluster randomized trial, Children, Right to health, Access to treatment, Standard of care, Kenya

## Abstract

Following the South African case, *Treatment Action Campaign and Others v Minister of Health and Others*, the use of 'pilot’ studies to investigate interventions already proven efficacious, offered free of charge to government, but confined by the government to a small part of the population, may violate children’s right to health, and the negative duty on governments not to prevent access to treatment. The applicants challenged a government decision to offer Nevirapine in a few pilot sites when evidence showed Nevirapine significantly reduced HIV transmission rates and despite donor offers of a free supply. The government refused to expand access, arguing they needed to collect more information, and citing concerns about long-term hazards, side effects, resistance and inadequate infrastructure. The court ruled this violated children’s right to health and asked the government to immediately expand access. Cluster randomized trials involving children are increasingly popular, and are often used to reduce 'contamination’: the possibility that members of a cluster adopt behavior of other clusters. However, they raise unique issues insufficiently addressed in literature and ethical guidelines. This case provides additional crucial guidance, based on a common human rights framework, for the Kenyan government and other involved stakeholders. Children possess special rights, often represent a 'captive’ group, and so motivate extra consideration. In a systematic review, we therefore investigated whether cluster trial designs are used to prevent or delay children’s access to treatment in Kenya or otherwise inconsistently with children’s right to health as outlined in the above case. Although we did not find state sponsored cluster trials, most had significant public sector involvement. Core obligations under children’s right to health were inadequately addressed across trials. Few cluster trials reported rationale for cluster randomization, offered post- trial access or planned to implement successful interventions. A small number of trials may have unnecessarily evaluated proven interventions, offered their control arm trial conditions worse than local standards of care or evaluated interventions ostensibly worse than local standards of care. Further research is required to establish if children’s right to health in cluster trials is well understood and to explain why some obligations are unmet.

## Introduction

Cluster randomized trials (cluster trials) are trials that randomly allocate interventions to intact groups or clusters of people such as schools or hospitals but collect information from individuals. In comparison, individually randomized control trials (RCTs) randomly allocate interventions directly to individual participants [1]. They are sometimes referred to as pilot studies, community based trials, knowledge translation trials and implementation trials.

Traditionally, cluster trial designs are used to assess effectiveness and feasibility of new interventions for specific settings, compared with RCTs, which assess efficacy of interventions. They are increasingly popular and important for sub Saharan Africa, where they can be used to address widespread and serious health challenges such as maternal and infant mortality, malaria, and HIV/AIDS, within severely restricted health resources, to develop interventions suitable for the environment.

Before publication of the Ottawa Statement on the ethical design and conduct of cluster randomized trials in 2012 (Ottawa Statement) [2], there was little other guidance and no international consensus on ethical or legal requirements for cluster trials conducted in this region. The Guidelines for the Conduct of Biomedical Research involving Human Research Subjects in Kenya (Kenyan Guidelines) published in 2004 cover all research involving participants but have no specific provisions for cluster randomized trials [3].

Yet, cluster trials raise unique issues which include, but are not limited to, inadequate reporting and rationale for cluster trials, incorrect identification of participants and cluster units, difficulties in ethics review and achieving informed consent, challenges with benefit harm assessment, and with protection of vulnerable participants, issues which are now receiving more attention [1,4-6].

Cluster trials in sub Saharan Africa encounter particular legal challenges associated with research in developing countries that largely remain unresolved and may be exacerbated by cluster trials and by the involvement of children. Key contested issues in this region include questions about justification for studies, standard of care offered by trials given prevalent low and unmet standards, and questions about the assessment of harms and benefits in marginalized or vulnerable communities, and about post trial access to successful interventions [7-10]. Host communities and stakeholders in the developing world are increasingly concerned with justice in research and the inevitable questions include how to equitably share the benefits and burdens of research and provide access to successful interventions [7-10]. As cluster trial designs are not well understood, ethical implications of children’s participation are not well understood and given the prevalence of ethical issues in sub Saharan Africa, children are particularly at risk of harm [11].

Children’s participation is a concern because they have a limited capacity to consent, rely on their legal guardians and thus may be exploited in cluster trials as in some cases, their legal guardians are not directly involved. In addition, children in sub Saharan Africa bear a heavy disease burden, their needs are highly prioritized and more cluster trials are likely to be designed and implemented to address their situation; this justifies a special examination of legal framework surrounding their participation and the use of cluster trials.

With individually randomized control trials in Kenya, research is justified when thresholds for equipoise are obtained; that is when there is genuine disagreement among the scientific community as to whether the new intervention is better than the available therapy, and when the benefits of participation are reasonable or outweigh the risks of participation for individual participants, and when control groups are offered similar or comparable standard of care that would be otherwise be available outside the study. All studies involving non-therapeutic interventions should minimize their associated risks consistently with sound scientific design and these risks should be reasonable in relation to anticipated gains [3].

Where children are involved, Kenyan Guidelines require studies to justify children’s inclusion and to seek consent from their legal guardian. Research where interventions are intended to offer direct benefits to the child should offer conditions to the control group that are as advantageous as available alternatives whereas studies not intended to offer therapeutic benefits, should involve minimal risk which should also be commensurate with the knowledge to be gained [3].

However, the above requirements, as well as other guidelines are difficult to interpret even in the context of RCTs, and their application to cluster trials involving children in Kenya is yet to be discussed. Little is known about the extent to which cluster trials involving children are used and why. The recent Ottawa Statement addresses some of the ethical issues raised by cluster trials, but have yet to be applied, adapted or discussed in the research context in sub Saharan Africa. The presence of vulnerable participants is noted as a special issue for cluster trials, but guidance on additional protection for vulnerable groups is left to researchers and ethics review committees [11].

Additional guidance can be drawn from children’s right to health, and would be compatible with current ethical frameworks for cluster trials as both Kenyan Guidelines and Ottawa Statement recommend that guidance for research and cluster trials should be read in light of local and international guidelines and law.

While children are one population among vulnerable populations requiring additional protection under ethical guidelines for research and under law, they are well recognized as a special group in Sub Saharan Africa and have a special right to health under local and international law, policy and guidelines for research which changes ethical requirements in cluster trials. Their special right to health is based on their vulnerability and weight and urgency of their health needs. Children in sub Saharan Africa are especially vulnerable because they face the very basic threat of survival (1). A recent World Health Report shows a third of all children are born in this region, and it is estimated that one out of nine children will die before they reach the age of five as compared to one in sixteen children in other regions, which is only a slight improvement over recent years [12].

Arguably, children’s claim to special protection has a strong moral impetus; a large number and significant proportion of the world’s population and Kenya’s population are at risk, they have little capacity to avoid or counter these dangers and their claim is more urgent as they face basic survival threats in comparison to other vulnerable adult populations in sub Saharan Africa who have survived their childhood.

The effect of children’s special right to health is that; they claim a larger share of resources and attention to address their circumstances, both in terms of healthcare and research with an emphasis on primary healthcare and services, and also this very basic threat to survival motivates their claim for special consideration and additional protection as expressed in numerous international and local law, instruments and policies [13-16].

Children’s claim for additional and special protection in the context of health and research, which are often in synergy with arguments for additional protection for vulnerable participants within ethical guidelines for research, are crucial for the ethical framework for cluster trials. They impose additional obligations on stakeholders involved in cluster trials and increase the protection for participant children and the wider population of children.

The implications of children’s special right to health in cluster trials are best expressed by the singular South African case of *Treatment Action Campaign and Others v Minister of South Africa and Others* (Treatment Action Campaign case) which was resolved by a South African Constitutional Court in 2002 [17]. The court looked into claims brought forward under children’s right to health on behalf of children outside a government 'research program’ who wanted access to an essential medicine restricted to pilot sites and questioned the justification presented by the government about the need for further research using pilot studies. This parallels concerns about trial conditions for control groups and access to successful interventions for the wider population as well as questions about the justification for cluster trials with children.

The case began when applicants led by Treatment Action Campaign, a local HIV/AIDS advocacy group, sued South African health authorities, arguing against their decision to offer Nevirapine only in pilot sites when evidence showed Nevirapine significantly reduced chances of transmission of HIV virus from mother to child, when donors offered five years free supply of Nevirapine as this was against children’s right to health and right to life as outlined in the South African Constitution and various other international instruments ratified by the South African Government including the United Nations International Covenant of Social and Economic Rights. The intervention in question, Nevirapine, involved a single dosage given to HIV positive expectant women during delivery as well a dose to the newborn child and this served to prevent HIV transmission from mother to child during childbirth. A large population was in need of this intervention and had no other alternative means to prevent HIV transmission to newborn children.

On the other hand, health authorities refused to expand access to Nevirapine beyond a few pilot sites arguing they were conducting further research and needed to collect more information about the effectiveness of offering Nevirapine on a wide scale and within a more comprehensive HIV/AIDS program and citing concerns about costs, feasibility of a program to provide substitutes for breast milk to prevent future transmission during early years, insufficient infrastructure, and possibilities that a wide scale resistance to Nevirapine could develop. The court ruled in favour of the applicants and the government appealed this decision at a South African constitutional court. In the Appeal, health authorities argued that the applicants wrongly sought to impose obligations on the government, which were primarily the responsibility of parents. A constitutional court dismissed this argument and government defence and held children’s constitutional right to health primarily imposed obligations on the Government in this instance. A similar constitutional claim for children’s right to health in other countries may impose obligations on the other governments.

The court ruled out pilot studies artificially restricting access to Nevirapine and asked the government to immediately expand access to affected children. This decision clarified the position on several issues including the acceptable rationale for cluster trials, the evidence gap of efficacy and effectiveness required for further research and particularly for cluster trials, trial conditions and standard of care to be offered to all arms, and post-trial access to beneficial interventions. The court found that benefits of Nevirapine far outweighed harms for affected children, as further delay would mean many more children born with HIV/AIDS. Efficacy of Nevirapine was established and acceptable as it significantly reduced chances of children being born HIV positive. The affected population had no other alternatives and faced a short life of suffering. Extra capacity to make Nevirapine available on a wide scale could be easily created if needed.

The claim for children’s special right to health was based on the South African bill of rights which provides in Section 27(1) that 'Everyone has the right to access to -… (a) health care services including reproductive health care’ and in Section 28(1) 'Every child has the right -… (c) to basic nutrition, shelter, basic heath care services and social services’ [18]. The Convention of the Rights of the Child and the International Covenant on Economic, Social, and Cultural Rights were also cited to support this interpretation of children’s right to health [14,19].

Parallels can be drawn with the situation in Kenya where children’s special right to health in Kenya was first outlined in Children’s Act of 2001 of Kenya, which provides in Section 3, 'The Government shall take steps to the maximum of the available resources with a view to achieving progressively the full realization of the rights of the child’ , in Section 4(1), 'Every child shall have the inherent right to life and it shall be the responsibility of the government and the family to ensure the survival and the development of the child,’ and in Section 9, 'Every child shall have a right to health and medical care, the provision of which shall be the responsibility of the parents and the Government’ [20]. In addition, Section 5 on non discrimination provides that 'no child shall be discriminated on’ [20].

In addition, Kenya has similarly ratified the Convention of Rights of the Child, which provides in Article 24.1 'State parties recognize the right of the child to the enjoyment of the highest attainable standard of health and to facilities for the treatment of illnesses and rehabilitation of health. State parties shall strive to ensure that no child is deprived of his or her right of access to such healthcare services’. Furthermore, in Article 24.2, state parties are obliged to take particular measures '(a) to diminish infant mortality’ and '(b) to ensure the provision of necessary medical assistance and healthcare to all children with an emphasis on the development of primary healthcare’ [14].

If these provisions are read together with the requirement in Kenyan Guidelines that research with children should provide participants with at least the available standard of care, the operative standard of healthcare for children is basic or essential healthcare [3.14,15,20]. This is reinforced by the long standing government policy of free basic healthcare for children under five [21].

Section 53(1)(c) of Kenya’s 2010 Constitution is more specific 'Every child has the right to the basic nutrition, shelter and healthcare’ and is almost *verbatim* to South Africa’s Constitution in Section 28(1) that “Every child has the right to basic nutrition, basic healthcare services and social services’ and this would affect cluster trials from 2010 going forward [22]. In comparison, adults are constitutionally guaranteed only the right to emergency medical care, although an argument could be made for women’s constitutional right to access reproductive healthcare and maternal healthcare in Kenya.

*Treatment Action Campaign and Others v Minister of Health and Others* set out the general foundation of children’s special right to health in the context of cluster trials and outlined some of the obligations. However, a more comprehensive reading drawn from the standard interpretation of human rights adopted in international documents, which defines three types of obligations for state parties, the duty to respect, protect and fulfill human rights, and more fully describes the obligations for government and other stakeholders involved in cluster trials [15,23].

In brief, the duty to respect the human right to health imposes a negative duty on governments to refrain from activities and actions that deny or limit equal access to the right to health for all parties. The duty to protect the right to health imposes an obligation on governments to take measures to ensure equal access to healthcare and health related services and this includes an obligation for government to ensure third parties do not limit equal access to healthcare and related services [15].

The duty to fulfill the right to health imposes an obligation on governments to progressively realize the right to health through legal, political systems and through a national health policy and provision of adequate healthcare services. The duty to fulfill and facilitate the right to health also includes an obligation to take positive measures to ensure individuals and communities enjoy the right to health. These types of duties will impose various obligations in cluster trials.

The duty to respect children’s right to health imposes a negative obligation on government not to use cluster randomized trial designs artificially to withhold access to healthcare from children in any arm. This obligation is stronger in the case of interventions offering essential or basic health care, where the delay results in grave consequences and where affected children have few other alternatives.

Under the duty to respect children’s right to health, governments are obliged to ensure children have equal access to healthcare and related services [15]. This rules out government sponsored cluster trials that do not offer trial conditions comparable to the local available standard of care, as this would discriminate against one group of children. As children are entitled to basic or essential healthcare under the Kenyan Constitution, Convention on Rights of the Child and General comment 14, they should be offered the available standard of care under the Kenyan Guidelines, ruling out rules out all cluster trials offering children less than the standard of care in any arm.

Furthermore, children’s right to health imposes an obligation on governments not to take regressive measures in relation to health unless these are deliberately taken and, in such cases, governments will have the burden of providing justification and showing due consideration [15]. This rules out cluster trials assessing interventions offering less than the standard of care without adequate prior justification and consideration.

The obligation to respect and protect children’s right to health rules out all cluster trials where there is sufficient evidence and consensus within the relevant scientific community about effectiveness and efficacy of the intervention in question, and where the needs of target children are pressing because such trials unnecessarily restrict or delay children’s access to healthcare, particularly where delays in implementation of effective interventions cause significant harm, such as in the above case where the intervention could have prevented HIV infection [15].

The duty to protect imposes an obligation on governments to prevent third parties from infringing children’s special right to health in cluster trials. This duty naturally falls on ethics review committees who are mandated by government to review cluster trials. Under this obligation and mandate, ethics review committees will generally ensure children’s special right to health is not violated in terms the prior evidence of efficacy and effectiveness presented by the protocol, the standard of care offered to control groups and access to successful interventions. In turn, under the duty to protect children, third party independent cluster trials and other organizations involved in cluster trials such as non-governmental organizations, charities, international sponsors, international development agencies and institutions will have a similar obligation not to violate children’s right to health and address aspects of standard of care, prior evidence of efficacy, and rationale.

Under the duty to fulfill children’s right to health, governments are obliged to provide, facilitate and promote children’s right to health, at a minimum of basic healthcare, adequate nutrition and shelter [15,22]. Thus governments are required to roll out interventions with proven efficacy, an obligation which is stronger when interventions provide basic healthcare and where the benefits are substantial and the risks faced serious or irreparable. This government obligation also applies to successful interventions assessed by independent cluster trials, which should be rolled out by government to wider populations of children with as little delay as possible, with priority given to those interventions offering basic healthcare to children.

The obligation to fulfill children’s right to health has implications on costs and feasibility of interventions. All cluster trials should consider viability of their interventions, assess cost elements and resources required, so as to minimize children’s research burden, maximize research opportunities and expedite access to healthcare. The obligation to fulfill also affects justification of cluster trials, some cluster trials are justified because they look into aspects of feasibility and effectiveness of proven interventions and, as such, their justification should clearly show that implementation is within reach, otherwise cluster trials involving children may be futile from a right to health perspective. Government sponsored cluster trials will have an obligation towards all children and should roll out successful interventions for the wider population while independent cluster trials have an obligation towards participating children and have a duty to roll out successful interventions within control groups. The obligation to fulfill children’s right to health also requires governments to promote and facilitate cluster trial designs where appropriate, as a means of progressively realizing children’s right to health.

In summary, children’s special right to health provides additional ethical guidance and clarification in areas that are traditionally problematic in research in resource restricted areas and with vulnerable populations. Cluster trialists are obliged to offer trial conditions equivalent to local standards of care, to assess prior evidence before mounting trials, and to provide access to treatment to control arms and to the wider population. Given concerns about misuse of cluster trial designs and the gap in the literature and guidance for cluster trials in this region, we conducted a systematic review to outline the use of cluster trials involving children to determine whether cluster trials in Kenya are used artificially to delay or limit children’s access to treatment, or otherwise designed and implemented to misinterpret or avoid obligations held under children’s right to health.

## Review

### Methods

Trials were eligible for review if published between 1 January 2003 and 28 February 2014, if publications reported using cluster randomization, if trials involved children aged between zero and eighteen, and if study sites were located in Kenya. The time period was selected to reflect human rights trends in cluster trials since Treatment Action Campaign was initiated in 2002, and also to account for CONSORT statement: extension to cluster randomized trials (CONSORT cluster statement), which published additional guidelines for cluster trials in 2004 [24]. Involvement is defined as contact with children either by collecting data specifically about children or administering or targeting interventions to children. Although expectant women were involved in the key case motivating this review, the intervention in question was primarily intended to benefit newborn children; the case was based on the government intention to deprive children of access to the intervention, made on behalf of children and the claim was successful based on children’s right to health. This review focused on cluster trials assessing interventions primarily targeting children; studies assessing interventions targeting only the health of expectant women or postnatal mothers (and not their offspring) were excluded. Studies that had only registered and not yet started collecting data or published their trial protocol by 28 February 2014 were excluded. Electronic searches were done on two key search locations, PubMed and ClinicalTrials.gov websites. Fourteen studies were eligible for review. Eligible studies were read and coded for each of the review measures below, information extracted and input into Table 1 by the first author. The second author reviewed this table and randomly sampled review measures and publications.

**Table 1:** Factors affecting children’s right to health in cluster randomized trials in Kenya, January 2002 to February 2014

**Author, Year of publication**	**Rationale for cluster randomization**	**Intervention**		**Standard of care pre trial**	**Evidence of efficacy and effectiveness**	**Results**	**Conclusions**	**Post trial access to intervention**	**Other involvement**
		**Intervention arm**	**Control arm**						
Ayieko *et al*., 2011 [25]	Reported reasons; logistically convenient and intervention targeted groups (pediatric teams)	Both interventions based on the same evidence-based best-practice guidelines.	Partial intervention:	All interventions above usual Government approach and practice, which is to adapt and distribute printed materials to staff and provide *ad hoc* training and opportunistic seminars	Intervention design based on 2004 study assessing delivery of care at first level referral hospitals in Kenya as well as theories from five publications suggesting approaches for improving pediatric care. Baseline data and several reports from Low Income	Outcome measures including 14 process and assessment tasks, and structure indicators. Most, not all indicators showed improvement, to varying degrees. For instance, higher completion of admission task, mean 0.94 versus 0.65, adjusted difference 0.54 (95% CI 0.05 to 0.29)	Conclusion: multifaceted intervention was effective but study advised against scaling up without further work on cost benefit analysis and because the system and resources may not immediately support intervention. For instance, no current role for facilitators or training program.	Guidelines are still available in public domain.	Strategy developed in partnership with the government
		Full intervention:	6 months surveys with only written feedback, provision of guidelines, job aides and 1.5 day seminar for 40 hospital staff.	None of the hospitals had explicit procedure for taking up new clinical care guidelines	Countries show low uptake of guidelines despite their availability slow uptake of guidelines common, sometimes linked to organization culture and change	Adoption of once daily gentamicin 89.2% versus 74.4 %; 17.1 (8.04 to 26.1%); loading dose quinine (91.9% versus 66.7%, 26.3 % (-3.66 to 56.3%); adequate prescriptions of intravenous fluids for severe dehydration 67.2 versus 40.6%; 29.9% (10.9 to 48.9%). Children receiving inappropriate drug doses lower in intervention hospitals quinine dose > 40%mg/kg/day (1.0% versus 7.5%; -6.5% (12.9% to 0.20%)). inadequate gentamicin dose (2.2% versus 9.0%; - 6.8% (-11.9% to -1.6%)		No changes announced on government approach.	Ministry of Health and Public Sanitation, district hospitals and pediatric teams involved in implementation
		a) three hospital assessments or survey done regularly every six months,	Similar job aids provided including a structured admission record to replace free standard notes	Admission records written as non-standard free text					
		b) Written and face to face feedback,			Other studies show financial incentives improve malaria specific outcome and implementation of guidelines improves pediatric emergency triage assessment, hospital care and outcomes for severe malnutrition.				
		c) three to five days of training aimed at hospital workers of all cadres, provision of guidelines, job aides,	Used alternative interventions (that is no placebo) for ethical reasons and because of uncertainty in literature about effect of multicomponent interventions to implement guidelines						
		d) An external supervisory process every two to three months							
		e) Identification of full time local facilitator to promote guidelines and onsite problem solving. One job aide for both groups was a structured admission record to replace previous free standing notes							
	**Rationale for cluster randomization**	**Intervention**		**Standard of care pre trial**	**Evidence of efficacy and effectiveness**	**Results**	**Conclusions**	**Post-trial access to intervention**	**Other involvement**
		**Intervention arm**	**Control arm**						
Brooker *et al*., 2010 [26]	Reported reasons: school clusters avoided contamination.	Arm 3 received both interventions	Arm 2) received only the literacy intervention,	Several unpublished surveys show 50% prevalence in malaria and 21 to 38% anemia in schoolchildren in region	Seven studies show school based intervention improves student achievement but few studies assess multifaceted interventions. Sri Lankan RCT showed malaria treatment improved exam scores. Previous Kenyan cluster trial showed reduced anemia and sustained attention but no effect on educational achievement. Multicentre study in eight sub Saharan African and Asian countries showed 50% of children in developing countries suffer from anemia. One systematic review showed anemia affected cognitive ability. Little experimental data for hypothesized causal chain: malaria prevention - reduced anemia - sustained attention - educational achievement	Of 88.3% children in intervention schools screened at baseline and follow-up. 17.5% tested positive	IST as implemented had no effect on health or education	In original protocol, schools in control arm were to receive literacy and malaria intervention after two years at end of study	The drug AL used in intervention identified through stakeholder consultations
	Inferred reasons: logistic convenience.	a) intermittent screening and treatment including rapid diagnostic testing every school term, AL treatment for positive tests	Arm 1) received only the malaria intervention						
	Study sought information on interactive effects of two interventions.	b) Literacy enhancement, training and support of class one teachers, teacher manual, workshops including weekly interactive texts and monthly written communiqués	Arm 4) did not receive any intervention	No school based malaria program. Educational achievement very low in region- district with worst mean scores in Kenya Certificate of Primary Education- national primary school matriculation exam and few children proceeding to secondary school.		81.8% in control and 83.0% in intervention group followed	Possible reasons: variable testing, reinfection, and geographical heterogeneity in transmission	However, researchers’ communication that final results were not positive, thus intervention will not be rolled out	Public randomization event involving community, stakeholders and researchers. Testing and treatment done by district health workers supported by the division of malaria control, MoHPS
	Both interventions school based intermittent malaria screening and treatment, and enhanced literacy instruction, required group delivery or use								
						No impact of malaria IST intervention observed on prevalence of anemia or malaria at either 12 or 24 months or on scores of classroom attention.			
						No effect of IST observed on educational achievement in older class, but an apparent negative effect noted on spelling scores in the younger class at 9 and 24 months and on arithmetic scores at 24 months			
				In 2009, GoK changed drug policy for malaria from quinine-based therapy to artemisinin combination therapy (ACT)					
	**Rationale for cluster randomization design**	**Intervention**		**Standard of care pre trial**	**Evidence of efficacy and effectiveness**	**Results**	**Conclusions**	**Post trial access to intervention**	**Other involvement**
		**Intervention arm**	**Control arm**						
Clarke *et al*., 2008 [27]	Not reported.	Interventions included: i) school based malaria prevention treatment- sulfadoxine pyrimethamine and amodiaquinine tablets 3 times a year (IPT)	Children received placebo tablets- no effect on malaria parasitemia, similar in size and shape but different in taste to intervention tablets	No information on school based programs malaria prevention and treatment of malaria.	Sri Lankan RCT showed school performance is related to cumulative effect of malaria attacks and reported weekly malaria treatment improved exam scores	Malaria prevalence in IPT group 6.3% and in placebo 12.6% adjusted risk ratio (0.51, 95% CI 0.29 to 0.93 *P* = 0.28).	School based IPT reduced prevalence of anemia and asymptomatic malaria in semi immune children and improved attention for school children but had no discernable effect on educational performance	School intervention not implemented	Primary schools and schoolteachers implemented study
	Inferred reasons: intervention delivered through a group-school								
				No Information on malaria and anemia: 2 studies showed 50% of children in region have asymptomatic malaria					
				Soon after this study implementation and before publications, GoK policy on first line malaria treatment changed from chloroquinine based drugs to ACT drugs	One review and one publication show lack of evidence to show malaria affects cognition and performance in school children. Three other African studies showed treating malaria in schoolchildren improved their cognitive abilities	Protective effect of IPT on anemia 48% (95% CI 8 to71, *P* = 0.028), *Plasmodium* prevalence lower in intervention than in placebo group 89% protective efficacy with 95% CI 73 to 95, *P* = 0.0002. Adjusted risk difference 0.35 (0.30-0.41). Cognitive tests and sustained attention	Effect on anemia was comparable or larger than effects produced through anti-helminthic or iron supplementation.	Government policy for malaria treatment changed and phased out the drug used for IPT in study	
	Logistical convenience	ii) passive surveillance of schools and local health systems for adverse reactions	Control group received items ii) to viii)				Study provided evidence of link between asymptomatic malaria and cognition		
					One report and one study say malaria chemoprophylaxis given to school children was associated with lower rates of malaria parasitemia and severe anemia, fewer clinical attacks and malaria deaths and reduced malaria related school absenteeism.	scores higher in IPT group			
							More work needed to replicate effect in different epidemiological settings, on operational feasibility, and on any long-term educational benefits of this approach		
		iii) one-day teacher training in social studies				Counting test adjusted mean difference 1.80 (0.19 to 3.41; *P* = 0.03), effect size 1.22 (95% CI 0.003 to 0.35) Code transmission test adjusted mean difference 6.05 (2.83 to 9.27; *P* = 0.0007).			
		iv) Finger prick blood samples; v) stool samples; vi) cognitive tests administered by psychologist; vii) social studies classroom tests administered by teachers; viii) end-of-term pupil assessment in social studies				No mean difference noted in hyper activity test or in educational achievement test			
	**Rationale for cluster randomization**	**Intervention**		**Standard of care pre trial**	**Evidence off efficacy and effectiveness**	**Results**	**Conclusions**	**Access to intervention**	**Other involvement**
		**Intervention arm**	**Control arm**						
Crump *et al*., 2005 [28]	Not reported.	Household based treatment of drinking water with flocculant disinfectant involving technology that removes suspended matter	Comparison group received Intervention 2: sodium hypochlorite solution	Source waters in study area have high fecal contamination and turbid water.	Guatemala RCT showed flocculant disinfection, (incorporating chemical used in municipal water treatment) can be used in household based water treatment	Flocculant disinfectant associated with significant reduction in diarrhea for children under two years old compared to local treatment practices	Further research needed to better understand association between water treatment and reduction in mortality	No reported provisions or arrangements for control arm or for all other arms after study	Not reported
	Inferred reasons:		Control group received intervention 3: Control - usual water collection treatment and storage, the use of safe storage containers not promoted	No health guideline for turbidity but WHO policy is five TBU for drinking water.	Bolivian, Zambian, Guatemalan and Uzbekistani studies show household-based water treatment reduces diarrheal diseases by 20 to 8%.	No significant difference in prevalence of diarrhea between interventions in all ages			
	intervention (a small water treatment facility) designed for use by households								
	Reduced contamination and			Study area-rural western Kenya had high turbidity ranging from 100 to 1000 TBU	Kenya study showed sodium hypochlorite less effective compared to flocculant disinfectant in highly turbid water and other study showed intervention less effective for pathogens resistant to chlorine	Data pooled from both arms showed a significant reduction in mortality in children < two years.			
	logistically convenient								
						Among people who have highly turbid source water, flocculant disinfectant can provide water that looks cleaner and reduces risk of diarrhea			
				Study reported most families collected water from ponds, rivers and springs using plastic containers and stored water in clay pots Families either treated water by					
					In children < two years, absolute difference in diarrhea prevalence compared to control: -25% in flocculant arm (95% CI -40 to -5) and -17% in sodium hypochlorite arm				
				decanting or cloth filtration, boiling, or chemical treatment of water not common					
					Effect in all age groups, absolute difference compared to control -19% in flocculant arm (-34 to 4) and -26% in sodium hypochlorite arm (-39 to -9). Fewer deaths in intervention compounds compared to control, relative risk ratio of death 0.58				
				No reported GoK standards for turbidity	*P* = 0.036			Sodium hypochlorite was commercially available in the market	
					*E. coli* concentration < 1 CFU/100ml 14% in intervention arm, 82% in flocculant disinfectant arm and 78% in sodium, hypochlorite arm. Mean turbidity 8 NTU, 55 NTU in other arms (*P* < 0.001)			At time of study, flocculant disinfectant for household use was not commercially available	
	**Rationale for cluster randomization**	**Intervention**		**Standard of care pre trial**	**Evidence of efficacy and effectiveness**	**Results**	**Conclusions**	**Post trial access to intervention**	**Other involvement**
		**Intervention arm**	**Control arm**						
Desai *et al*., 2004 [29]	Not reported.	Arm I: daily supervised iron supplementation	Arm II: daily unsupervised iron supplementation	Study reported lack of standardized guidelines for iron supplementation Previous study reported iron supplementation was not routine for children with mild to moderate anemia and was prescribed in only 12% of children diagnosed with severe anemia who also received presumptive antimalarial treatment.	Several studies showed weekly or biweekly supplementation is as efficacious in prevention and treatment of mild to moderate anemia as conventional daily supplementation despite 30 to 70 % reduction in cumulative dose	In supervised groups hemoglobin concentration was higher in the daily iron group than in the twice-weekly iron group.	For this region, and after initial antimalarial treatment, daily iron supplementation is better than twice weekly iron supplementation for hemoglobin level regardless of whether adherence can be ensured	No information	Not reported
	Inferred reasons: logistically convenient to randomize children by the compounds they lived in,								
						Mean difference at 6 weeks 4.2 g/L (2.1 versus 6.4) and at 12 weeks 4.4 g/L (1.8 versus7.0) with 95% CI			
			Arm III: twice weekly supervised iron supplementation	Clinics that prescribed iron for severe anemia use short courses involving two weeks of relatively high doses of iron, which is combined with a presumptive antimalarial treatment	However, a 1999 meta- analysis showed daily iron supplementation compared to intermittent supplementation was more efficacious for pregnant women, the beneficial effect in preschool children is inconclusive and indicated that the degree of supervision affected anemia prevalence. A 1999 study with aboriginal communities showed six weeks of unsupervised twice-weekly iron supplementation superior to unsupervised daily supplementation for increasing hemoglobin in children				
						Among unsupervised groups, hemoglobin concentration did not differ at 6 weeks mean difference 0.86 g/L (-1.4, 3.1) but were higher at 12 weeks for daily iron group, mean difference 3.4 g/L (0.79 versus 6.0) *P* = 0.02 95% CI			
	reduced contamination between study arms		Arm IV: twice weekly unsupervised iron supplementation					Additional benefits of participation - health passports issued to all children in trials to allow free healthcare at local clinics and at hospital	
	**Rationale for cluster randomization**	**Intervention**		**Standard of care pre trial**	**Evidence of efficacy and effectiveness**	**Results**	**Conclusions**	**Post trial access to intervention**	**Other involvement**
		**Intervention arm**	**Control arm**						
Freeman *et al*., 2012 [30]	Not reported.	Full Intervention only given to Arm 2: hygiene promotion (HP), water treatment supplies (WT), and three-day training of teachers in hygiene promotion (HP), behavior change and water treatment with regular follow-up visits in school year	Arm 1: hygiene promotion and water treatment supplies (hand washing and drinking water containers and one time supply of disinfectant known as water guard).	GoK ratio for latrines in schools is 25:1, one latrine for every 25 girls and 30:1, and 1 latrine for every 30 boys	Three studies show school based hand washing or water treatment in low-income settings reduces school absence by between 21 and 42%	HP and WT intervention no significant impact on absence, OR 0.81, 95% CI 0.50 to 1.35 even when sanitation was added	Improved WASH access did not mitigate absence on boys,	Arm 3 received all interventions after study	Program embedded within a larger research and learning project run by CARE (an international NGO).
	Inferred reasons: school based water treatment, sanitation and hygiene promotion intervention intended for delivery through groups-schools.								
			Three-day training of teachers on hygiene promotion, water treatment, but no additional latrines						
			Intervention in Arm 3: control group received no intervention during study,			OR 0.63, CI 0.31 to 1.27.			
		Schools received hand washing and drinking water containers and one time one-year supply of water guard (1.2% chlorine based water disinfectant promoted by Population Services International (PSI)			One systematic review shows WASH cluster trials in middle-income countries had limitations (small sample sizes/non-adjustment for school level clustering/utilization of non- equivalent group design). Most frequently cited evidence on impact of WASH is from a non-experimental study with 11% reduction in girls’ absence in Bangladesh and included monetary subsidies.		Several factors affected study; heterogeneity among schools, election violence and displacements resulted in schools closing for four months		
					One systematic review, a combined systematic review and meta-analysis, one quantitative systematic review shows WASH at home improves health of children under five years old				
		Provision of latrines to GoK standard to maximum of seven latrines, not more than seven latrines proved to any one school.							
							No significant difference between single and multiple WASH interventions		
							No impact noted on enrollment or test scores		
				Schools selected for study had to exceed GoK ratio.					
			All groups received deworming after baseline and once more during study	Only 29% of schools in study area met the government ratio		However, both interventions reduced absence in girls although the study did not know how.			Study area chosen through rapid assessment by study partners, study partners not named likely includes the Kenya National Bureau of Statistics.
						WASH/HP and WT alone reduced the odds of 2 week school absence in girls by 58% (OR 0.42, 95% CI 0.21 to 0.085 but no effect on boys (OR 0.88, 0.45 to 1.71).			
						WASH, HP and WT and sanitation showed comparable benefit for girls (OR 0.47, .21 to 1.05) but no benefit for boys (OR 0.98 95% 0.52 to 1.87).			
						For HP and WT 0.34 days of absence were avoided per pupil per two week recall period and			
						For HP, WT and sanitation 0.38 days			
	**Rationale for cluster randomization**	**Intervention**		**Standard of care pre trial**	**Evidence of efficacy and effectiveness**	**Results**	**Conclusion**	**Post trial access to intervention**	**Other involvement**
		**Intervention arm**	**Control arm**						
Gewa *et al*., 2013 [31]	Not reported.	Arm 1: vegetarian	Arm 4: control group no food supplementation	Not reported.	Guatemalan study showed that in household based programs, foods can be redistributed to non-targeted household members, and studies in Bangladesh, India and Philippines showed daily energy intake rose about 75 to 100% on school feeding days. No studies measuring effect of school based feeding program on distribution of food in households	Children in meat group had increased energy and protein intakes at follow- up but these changes were not significantly different from changes experienced by control group. Children in vegetarian, milk and control group did not experience significant changes in energy and protein intake.	No evidence that school children who received supplementary feeding at school experienced reduced intakes at home or that food intake for other family members was increased at the expense of school children	Not reported	Project implemented by Child Nutrition Program in turn supported by a grant by United States Agency for International Development (USAID)
	Inferred reasons: cluster randomization logistically convenient and reduced contamination between arms								
	Intervention intended for delivery through groups-schools								
		A serving of maize and bean (*gither*i)		No official school feeding programs to cover all primary school going children but there may have been independently sponsored programs by parties such as churches, self help groups, parent-groups or non-governmental organizations (NGOs)					
						Parents: significant decline in protein intake reported for parents of children in vegetarian group. Other three groups did not experience significant decline			
		Arm 2: *githeri* and meat serving Arm 3: milk and *githeri* dish							
	**Rationale for cluster randomization**	**Intervention**		**Standard of care pre trial**	**Evidence of efficacy and effectiveness**	**Results**	**Conclusion**	**Post trial access to intervention**	**Other involvement**
		**Intervention arm**	**Control arm**						
Kangwana *et al*., 2011 [32]	Not reported.	Retail outlets in sub locations in intervention arm received subsidized pediatric Artemether Lumenfantrine (AL) and one day training for retail staff.	No intervention provided, retailers did not receive subsidized AL	AL first line recommended treatment for uncomplicated malaria, available only through prescriptions and at no cost in public facilities	Pilot programs in Tanzania and Uganda show rapid uptake of subsidized ACT, decreased use of old regimens and good adherence to prices. Senegal study showed irregular availability, Cambodia study showed high prices. Multi country study using community distributors showed increased uptake of AL	Percentage of children receiving AL in same or following day in intervention arm was 35.9%, higher than those receiving AL in the control arm 14% by percentage difference of 25% points 95% CI, *P* = 0.0002, adjusted *P* = 0.0001.	While increase in prompt ACT coverage rose to 44.9% it is still well below roll back malaria targets, and more work is needed to understand ACT subsidy mechanisms and effects, on effect of enhanced diagnosis and cost and effectiveness studies on public sector strategies versus community driven approaches	Study did not roll out intervention.	Pharmacy and Poisons Board of Kenya (PPB), Population Services International staff collected complete referral forms from retail outlets. PSI is an international NGO with presence in many developing countries
			In 2006 to 2007 the government conducted AL awareness campaigns, so both arms received some general info on current malaria policy						
		Community awareness: nine community leader workshops, ten small group discussions targeting two hundred people and outreaches by community based organization targeted 21,000 people, branded t-shirts, pens and scarves given to community, shops given posters or painted for advertisement							
				In practice often sold without prescription, private sector retail price 500 Kenyan shillings or 6.16 USD					
						Tibamal, the intervention drug, constituted 63% of all ACT used.			
				Old antimalarial regime was still being sold at 0.37 USD on average.					
					At time of study, ACT subsidy mechanism AMF-m was in development Global Fund to make direct payments to prescribed manufactures to lower import cost to public and private retailers				
	Inferred reasons:			Frequents stock outs of AL reported in public facilities.		93 % (SD 5.9%) of Tibamal was bought at recommended retail price 0.25 USD no significant difference in accuracy of dosage was recorded between intervention and control group	Study highlighted likely differences between this intervention and the roll back malaria (RBM) program where AL is usually availed through prescriptions and in health facilities.	Increased coverage of AL achieved through other means, at follow -up: 65% of GoK health facilities stocked AL.	Implemented by Division of Malaria, MoHPS and PPB
	to avoid contamination			WHO report: only 15% of children with fever < 5 years in malaria endemic Africa promptly received Artemisinin- based combination therapy (ACT)			Over 30% accessed AL through retail sector but		
	among participants.						increased public sector coverage noted during study.		
	Involved group level delivery of intervention								
	**Rationale for cluster randomization**	**Intervention**		**Standard of care pre trial**	**Evidence of efficacy and effectiveness**	**Results**	**Conclusion**	**Post trial access to intervention**	**Other involvement**
		**Intervention arm**	**Control arm**						
Opondo *et al*., 2011 [33]	Not reported.	Intervention comprised of multifaceted implementation of MoH adapted guidelines to reduce inappropriate use of antibiotics: training on guidelines, supervision, and support, including face-to-face feedback.	Control arm received partial intervention which included adapted guidelines, didactic training,	Guidelines are usually adapted by MoH and sent to hospitals in written format, or charts, *ad hoc* training is provided.	Guideline well established and recommended by Integrated Management of Childhood Illness and WHO guidelines.	Multifaceted implementation of guidelines reduced the inappropriate use of antibiotics in non-bloody diarrhea.	Intervention to reduce inappropriate use of antibiotics when nested within a large integrated approach will not only significantly reduce inappropriate use but also improve pediatric care in other areas. Intervention effects may cut across other areas in care.	No information provided but parent study did not roll out intervention.	Study based on analysis of data previously collected by Ayieko *et al*., 2011 which examined uptake of 14 point pediatric admission guidelines for rural district hospitals.
		Guidelines for diarrhea alone include taking history, assessing the patients for shock and dehydration, classification of severity of dehydration and appropriate rehydration therapy as well as other four points.							
	Inferred reasons: study emphasized intervention is intended for hospital level, logistically convenient and group effects useful		Written feedback, facilitation and supervision						
						9,549 admission records reviewed, 4,232 diagnosed with diarrhea and 1,160 diagnosed with non-bloody diarrhea. 750 children received antibiotics inappropriately, 313 in intervention hospitals and 437 in intervention hospitals. Odds of children receiving inappropriate antibiotics for non-bloody diarrhea in intervention hospitals was 0.30 that in control hospitals (95% CI 0.09 to 1.02)			Study implemented in district hospitals, Staff involved, cooperation from MoH, MoHPS
				No information provided on adherence to or uptake of guideline					
						(95% CI 0.09 to 1.02)			
				Guidelines: for patients with diarrhea and non-bloody stool, manage with fluids and for patients with bloody stool, give antibiotics					
					Botswana study shows bloody diarrhea is often caused by *Shigella* and made worse by antibiotics. One study showed antibiotic misuse increases antibiotic resistance. Botswana study showed non-adherence to guideline is common.				
					Literature from Niger, South America, Pakistan and Armenia show implementation of diarrhea management guideline difficult				
	**Rationale for cluster randomization**	**Intervention**		**Standard of care pre trial**	**Evidence of efficacy and effectiveness**	**Results**	**Conclusion**	**Post trial access to intervention**	**Other involvement**
		**Intervention arm**	**Control arm**						
Patel *et al*., 2012 [34]	Not reported	Thirty villages	Thirty villages 405 students 331 households 22 schools	Study did not publish information on standard of care. Many primary schools in the area may not have access to treated water and study did not provide information on government arrangements to provide soap or water in public primary schools. There may be some hygiene education within primary school curriculum.	Two systematic reviews showed handwashing with soap reduced risk to both illnesses and household based water treatment also shown to reduce diarrhea risk.	Mixed findings: percentage of students who could demonstrate proper handwashing technique was higher in intervention arm at mid point (46% versus 14% EDM 32%, 90% CI 10 to 46%), but similar at second follow-up (54% versus 50% EDM 0% 90% CI -17 to 11%).	Handwashing stations and hygiene education may have contributed to reduced rates of illness in primary school children but changes did not result to reduced diarrhea rates, study authors attributed this to the effect of other interventions being run in that region	One year later intervention rolled out to control schools	Project situated within a wider Nyando Integrated child health and education program in the region
	Inferred reasons: logistically convenient to randomize by schools, reduced contamination and measurements of group effects were useful								
		378 students,				At first round reduction in percentage of students with any illness (5% versus 7% EDM -3%, 90% CI 4 to 1%) and with ARI (2% versus 3%, EDM -2%, 90% CI -3% to -1%) but similar rates at second round for ARI (0.8% versus 0.7%, EDM 0%, 90% CI -1 to 1%) or any illness (3% versus 2%, EDM 1%, 90 CI -1 to 1%).			
					Studies in Ghana, Oudomxay and western Kenya showed school based programs increase hygiene knowledge among students and changed practices. Four studies in Kenya and one systematic review showed WASH programs reduce absenteeism but studies have not shown a direct impact on health.	'No difference noted in diarrheal illness between intervention and comparison arm during either year of surveillance (year I: 0% versus 0.3% EDM (0% 90 CI 0 to 0%; year II 0% versus 0% EDM 90% CI 0 to 0%)			
		312 households							
		21 schools.							
		Teachers in intervention schools received handwashing and water treatment training and teachers provided instructional material for students. Schools received drinking water stations and handwashing stations as well as three month starter supply of soap and water treatment solution. Home visits and school visits in both arms to collect data							
	**Rationale for cluster randomization**	**Intervention**		**Standard of care pre trial**	**Evidence of efficacy and effectiveness**	**Results**	**Conclusion**	**Post trial access to intervention**	**Other involvement**
		**Intervention arm**	**Control arm**						
Philips-Howard *et al*., 2003 [35]	Not reported	113 households were given	No bed-nets for duration of study	Not reported	RCTs in Ghana, Gambia, Kenya and Burkina Faso showed insecticide treated bed- nets to be effective in reducing all cause mortality in children under five years by 17%	Crude mortality rates were 51.9 in control village compared to 43 in intervention villages per 1,000. The protective efficacy with 95% CI was 16% (6 to 25%).	Treated nets prevent approximately one out of four deaths in areas with high perennial malaria transmission but are not as effective if retreatment is delayed beyond six months	Villages in control arm received intervention after study	Study funded by USAID
	Inferred reasons: study wished to measure effect of the intervention on the group.								
	Intervention intended for public health level administration,					Protective efficacy of retreated nets overall 20% (10 to 29%), and 26% (12 to 37%) in children aged between one and eleven months old children and 14% (-1 to 26%) in children aged between twelve and fifty-nine months old			
				Study conducted in between 1997 to 1999, did not report information on access to bed-nets for young children within national malaria program, but treated bed-nets may not have been part of malaria program					
					A large-scale social marketing program in Tanzania estimated a 27% increase in survival of children under five but there is scientific disagreement whether treated bed-nets reduce mortality in very young children in areas with high transmission pressure				
		Treated bed-nets to cover sleeping space and the nets retreated after six months							
	logistically convenient,								
	reduced likelihood of contamination among participants								
	**Rationale for cluster randomization**	**Intervention**		**Standard of care pre trial**	**Evidence of efficacy and effectiveness**	**Results**	**Conclusions**	**Post trial access to intervention**	**Other involvement**
		**Intervention arm**	**Control arm**						
Skarbinski *et al*., 2009 [36]	Not reported	Study provided rapid diagnostic tests (RDTs) to all health facilities	Training: one-off three day training to all health facilities.	GoK to implement new malaria policy between April and October 2006: deliver AL to all health facilities, provide training on new malaria guidelines and on use of AL and diagnostic tests at least one per facility	Three studies in Zambia and one study in Kenya showed health workers under-prescribed AL and over- prescribed other antimalarial drugs	Treatment: RDTs significantly reduced recommended treatment by 63% (*P* = 0.04)	Providing RDTs significantly reduced recommended treatment without reducing overtreatment	No arrangement in protocol to provide RDTs to comparison arm	No information.
	Inferred reasons: intervention, rapid diagnostic tests, intended for groups - pediatric teams and health facilities receiving, diagnosing and treating pediatric malaria								
				At baseline, 25% of health facilities in study region received RDTs from GoK					
			Artemether Lumenfantrine			TGS alone increased recommended treatment by 41% (*P* = 0.05).		Increase independent of trial. By time of study implementation, 50% of patients in each arm were seen in facilities where RDT was available	
						Neither the RDTs or microscopy significantly reduced overtreatment with non-recommended antimalarial (prescribed old antimalarial for patients who tested positive)			
				Drug SOC:	AL - new but expensive first line treatment for un- complicated malaria	Testing: in intervention arm use of RDTs increased from 35% to 46%, use of blood slides decreased from 38% to 8% but overall use of tests (RDTs or microscopy) did not change			
				At time of study most facilities received AL but routine in service training incomplete.					
				Testing SOC: limited lab capacity & only 25% of health facilities had received RDTS through the Government	June 2006 - GoK introduced AL as recommended first line treatment of uncomplicated malaria, revised national malaria treatment guidelines and a new treatment algorithm				
		Training: one-off three day training to all health facilities.	A copy of the revised national malaria treatment guidelines,			Adherence to treatment guidelines: RDT significantly reduced use of clinical diagnosis of malaria to prescribe AL by 36% (*P* = 0.03)	Because RDT use replaced microscopy and provision of RDT reduced proportion of AL prescriptions for patients who were not tested		Implemented in government health facilities- hospitals, health centers, dispensaries,
		Artemether Lumenfantrine	Supervision (TGS)				More work is needed on health worker approach to RDT use and antimalarial use. Several reasons put forward by study: conflicting messages in TGS		
		A copy of the revised national malaria treatment guidelines							
		Supervision							
		(TGS)							
	**Rationale for cluster randomization**	**Intervention**		**Standard of care pre trial**	**Evidence of efficacy and effectiveness**	**Results**	**Conclusions**	**Post trial access to intervention**	**Other involvement**
		**Intervention arm**	**Control arm**						
*Suchdev et al*., 2012 [37]	Not reported.	In intervention villages, sprinkles sachets (branded MNPs) socially marketed and sold to households with children 6 to 59 months by women trained by safe water and AIDS project (SWAP)	Control arm: no MNPs sold. Other SWAP products such as soap, insecticide treated nets, water disinfectant and condoms sold in all arms	Public health authorities distribute vitamin A in health facilities	One Cochrane review, one systematic review and meta -analysis show MNPs reduce anemia, have higher uptake and fewer side effects than iron drops	27.2% absolute reduction and 40.9% relative reduction in prevalence of anemia in intervention group and 20.1% absolute and 29.9% relative reduction in control group and *P* = 0.10	Community based distribution of MNPs sprinkles improved recovery rates for anemia and some measures of iron and vitamin A deficiency even where children received less than recommended doses of iron	After follow-up survey, community based distribution of MNPs expanded to cover control villages	
	Inferred reasons: intervention (micronutrients powders-MNPs) delivered through a group mechanism - a community program that that trained women to distribute MNPs.								
						Decrease in prevalence of iron deficiency by 19.3% in intervention group compared to 5.3% in control, *P* = 0.001			
	Study a pilot as sponsors and partners intended to increase coverage within the wider community project								
						Vitamin A deficiency decreased by 7.5% in intervention and 2.5% increase in control (*P* = 0.01)			
					One double blind placebo-control trial compared doses and offered free MNPs found anemia recovery rate of 53%				
				MNPs also supplied by authorities, oral dosing may also be used					
		Cost two Kenya shillings per sachet (approximately 2.7 US cents at the time)		Government of Kenya (GoK) changed policy in 2007 from mass distribution of vitamin A with 80% cover in study region, to provision in health facilities resulting to 22% cover	Bangladesh, Mongolia and Bolivia have national program, other countries planning large-scale programs				Sprinkles global health initiative provided MNPs
					One community based cluster randomized cluster trial showed many disadvantages and low adherence to prophylactic micronutrient supplements		Study had recovery rates similar to previous clinical trials where MNPs distributed at no cost, thus approach is potentially self-sustaining		SWAP: Safe Water and AIDS Program trained the vendors who were women from the community
	**Rationale for cluster randomization**	**Intervention**		**Standard of care pre trial**	**Evidence of efficacy and effectiveness**	**Results**	**Conclusions**	**Post trial access to intervention**	**Other involvement**
		**Intervention arm**	**Control arm**						
Zurovac *et al*., 2011 [38]	Not reported.	Between Monday to Friday, one-way text messages about malaria case management sent to health workers’ personal mobile phones for period of six months	No intervention provided.	No previous information on health workers’ adherence. Studies in Angola, Uganda and Tanzania and one publication on sub Saharan Africa found despite simple guidelines for managing malaria in children with fevers, there is little health worker adherence to case management, prescription, dosing, counseling or dispensing practices	Researchers conducted a systematic review and assessed strategies to improve health worker performance and found an overall median improvement of 9% (IQR 3 to 23%)	When all 4 treatment tasks and 5 of 6 dispensing and counseling tasks were measured, improvement of 21.4% points in short term (95% CI 9.0 to 33.7, *P* = 0.0007), and 23.7% (11.6 to 35.7, *P* = 0.0001) after six months.	Intervention worked just as well and better than more complex interventions, and achieved modest adherence of 9 %	No reports of arrangements	Division of Malaria at Ministry of Health and Public Sanitation assisted in intervention development
	Inferred reasons: intervention targeted health workers in health facilities,								
		Messages extracted from national guidelines and training manuals		At baseline, study found 9 % adherence to treatment guidelines for outpatient pediatric malaria					
	logistical convenience								
						Effect size smaller when study measured all 4 treatment tasks and all 10 dispensing and counseling tasks at short term 10.3% (4.0 to 16.6, *P* = 0.0013) at 6 months and 11.3% (5.1 to 17.6, *P* = 0.0004) at 6 months			
					Two Kenyan RCTS show concept works, texts sent from health				
		Texts included short motivating/entertaining quotes	Surveys conducted at baseline and follow-up to measure adherence to treatment guidelines.		workers to HIV/AIDS patients improves patient adherence to treatment regimen		Study did not understand why/how text messaging health workers worked	Study undertaking a cost and operational assessment of their trial with the government looking at how to replicate on national scale	
		Cost of single text 0.01 USD, full exposure in intervention cost 2.96 per health worker	All arms received three rounds of malaria case management training sessions for health workers as well as national guidelines, drug management wall charts for ACT		Kenyan RCT using in-service training and passive job aide distribution to improve health workers’ malaria case management showed slight improvement		More work on cost effectiveness of study.		
					Two RCTS in Angola and Uganda investigated texts to health workers and looked at short-term effects		Despite low cost of texts more funds may be needed to establish and maintain the system.		
							Researchers recommended intervention to supplement other adherence strategies		

Columns of results and conclusions are separated in this table to improve readability as some cluster trials reported complex results and some large cluster trials reported a larger amount of data.

*Abbreviations:* AL: Artemether Lumenfantrine; ACT: Artemisinin based combination therapy; AMF-m: Affordable Medicines Facilities-Malaria; CARE: Cooperation for Assistance and Relief Everywhere; CDC: Center for Disease Control, United States; CI: confidence interval; GoK: Government of Kenya; HP: hygiene promotion; HW: health worker; IMCI: Integrated Management of Childhood Illnesses guidelines; IPT: intermittent preventative treatment, for malaria; IST: intermittent screening and treatment, for malaria; Kshs: Kenyan shillings; KEMRI: Kenya Medical Research Institute; KNH: Kenyatta National hospital, main referral and teaching hospital in Kenya.; LIC: low income countries; LSHTM: London School of Tropical Health and Medicine; MNP: micro nutrient supplement powder; MoHPS: Ministry of Health and Public Sanitation; MoE: Ministry of Education; p: p value; PPB: Pharmacy and Poisons Board; PSI: Population Services International, an international health charity; NGO: non-governmental organization; RBM: roll back malaria program; RCT: randomized control trials; RDT: rapid diagnostic test kit, for malaria; USAID: United States of America International Development; USD: United States dollar; WASH promotion: water and soap provision and hygiene promotion.; WHO: World Health Organization; WT: water treatment.

### Review measures

Review measures are factors that affect the right to health raised by the design and implementation of cluster trials involving children and were developed and validated by the first and second authors. These measures are:

1) Rationale for cluster trial design

2) Interventions

3) Pre trial standard of care

4) Prior evidence of efficacy and effectiveness

5) Results and conclusions

6) Post-trial access to interventions

7) Government involvement

With the CONSORT cluster statement, cluster trials are required to report their rationale for using cluster randomization and only a narrow range of circumstances are ethically acceptable. Rationale can also indicate whether cluster trial designs are misused, whether ethical implications are well understood and requirements under children’s special right to health will follow from the study rationale. Under children’s special right to health, cluster trials are required to offer a comparable standard of care to that available in the region, and where there are questions about accessibility of stated standards, cluster trials are required to show prior justification for developing a standard of care lower than available standards and to show that government had duly considered this intervention and the justification to offer this. This may include evidence of unmet standards.

Cluster trials are also required under children’s special right to health to show any prior evidence of efficacy and effectiveness of their interventions to show that clinical equipoise had obtained in the trial and further research was warranted. The results and conclusions of the cluster trials indicate the end point of trials, which allow for a retrogressive review of cluster trial rationale, and for further review of designs. In addition, children’s special right to health imposes obligations on governments to implement cluster trials in the wider population and to implement positive results in control arms. Government involvement in cluster trials is an important indicator of where obligations should fall.

## Results

Table 1 Factors affecting children’s right to health in cluster randomized trials in Kenya between January 2002 and 28^th^ February 2014.

Fourteen cluster trials were conducted in Kenya involving children and published between January 2002 and September 2013. Gewa et al. [25] was published in 2013. Three studies, Patel et al. [26], Suchdev et al. [27] and Freeman et al. [28] were published in 2012. Four studies, Opondo et al. [29], Zurovac et al. [30], Kangwana et al. [31] and Ayieko et al. [32] were published in 2011. The remaining six studies were published before 2010 and as far back as 2003, including Brooker et al. [33], Skarbinski et al. [34], Clarke et al. [35], Crump et al. [36], Desai et al. [37] and Philips-Horward et al. [38].

Six out of fourteen cluster trials looked into various aspects of malaria. Four studies involved only children and of these, two studies focused solely on malaria. Kangwana et al. assessed retail sector delivery of subsidized Artemether Lumenfantrine for treatment of malaria in children aged between three and fifty-nine months [31]. Zurovac et al. evaluated phone-text support to health workers as a means of improving adherence to guidelines for malaria diagnosis and treatment [30]. The other two studies investigated a combination of health issues. Brooker et al. investigated whether malaria screening and treatment combined with enhanced literary instruction in primary schools could reduce malaria related anemia and increase educational achievements, [33] and Clarke et al. looked at whether an intervention delivering malaria preventative treatment programs combined with education instruction in schools could reduce malaria related school absence, anemia and improve education performance in school children [35].

Two of the six studies focusing on malaria provided their intervention to both adults and children. Skarbinski et al. assessed the impact of providing rapid diagnostic testing kits in health facilities on the overall rate of diagnosis and treatment of malaria in patients over the age of five years, [34] and Philips-Howard et al. assessed the impact of providing treated bed-nets to communities on mortality in infants less than 60 months old [38].

Three out of fourteen cluster trials focused on nutrition. Gewa et al. investigated whether providing food supplementation to children in primary school led to reallocation of food at home [25]. Suchdev et al. piloted a community based program distributing micronutrient supplements targeting children between six and thirty five months old to address anemia, [27] and Desai et al. tested daily iron supplementation compared to other modes for reducing anemia in children less than five years of age [37].

Three out of fourteen cluster trials evaluated water treatment, sanitation and or hygiene interventions. Two of these three studies assessed school based interventions: Patel et al. assessed the impact of an intervention providing a hygiene curriculum, handwashing and drinking water stations in schools on hygiene practices, diarrhea and acute respiratory illness, [26] and Freeman et al. evaluated whether providing water, sanitation, hygiene promotion in schools affected school attendance [28]. The remaining study, Crump et al., investigated the effect of household based flocculant disinfection of water on mortality and diarrhea in children less than two years of age in areas using water from turbid sources [36].

Two of out of fourteen cluster trials focused on pediatric guidelines in health facilities. Ayieko et al. evaluated whether multifaceted implementation of guidelines in rural district hospitals improved pediatric care, [32] and Opondo et al. was a secondary analysis of data focusing on a specific guideline to reduce inappropriate use of antibiotics to treat non-bloody diarrhea in children [29].

### Rationale for cluster trial design

Only two out of fourteen cluster trials clearly reported their reason for using cluster trial design. Ayieko et al. assessed an intervention providing multifaceted implementation of pediatric care guidelines in district hospitals and reported their intervention was intended for groups so cluster randomisation was logistically convenient [32]. Brooker et al. evaluated the effect of intervention providing intermittent malaria screening and treatment combined with enhanced literacy instruction to reduce malaria related anemia. Brooker et al., used school clusters to reduce contamination among participants [33]. Cluster trials are often used instead of traditional trials to create distance between the subjects of the trials to reduce the possibility that members of one cluster adopt the behavior of members in the comparison cluster by 'contamination’ and so damage the results. The remaining twelve cluster trials did not clearly report why they used cluster randomization. However, we inferred from published information in all cluster trials that cluster randomization was logistically convenient and would reduce contamination.

### Standard of care before trial

a. **All trial arms receive conditions comparable to standard of care**

Ten of fourteen studies offered their intervention arms a standard of care similar to or better than the government’s stated regional standard, while clusters in their control arms received the stated regional standard of care.

Gewa et al. offered a school based feeding program in three arms, while children in one arm received the regional standard which was not to receive food supplementation [25]. Patel et al. offered a drinking water, handwashing and hygiene promotion intervention in intervention schools, where the regional standard was no intervention, which served for control schools [26].

Ayieko et al. and Opondo et al. offered a full multifaceted implementation of pediatric guidelines in rural district hospitals including hospital assessments, longer training, more supervision, face-to-face feedback and support from a facilitator [29,32]. Control arm hospitals received an active control involving adapted guidelines, less training, written feedback, less facilitation and less supervision. The active control was still better than the government approach to implementing guidelines, which relied on opportunistic training and mainly adapted written guidelines.

For their intervention arm, Zurovac et al. sent daily text message reminders to health workers containing key points of guidelines on pediatric malaria diagnosis and treatment [30]. Health workers in the control arm did not receive any intervention, which was the regional standard of care.

Brooker et al. offered schools in the intervention arm enhanced literacy instruction and malaria prevention and treatment, neither intervention was available in the region [33]. Schools in the control arms received either the literacy component, or intermittent screening and treatment component, or did not receive any component of the intervention.

Skarbinksi et al. offered rapid diagnostic test kits for malaria to health facilities in their intervention arm [34]. All arms received training, guidance and supervision on how to use rapid diagnostic kits, the new national malaria protocol as well as training on the use of the protocol. Health facilities in the control arm did not receive extra kits from the study, but continued to offer patients the regional standard of care, which was eventually to have at least one diagnostic equipment item per health facility.

Clarke et al. offered schools in their intervention arm intermittent malaria prevention to pupils and one-day teacher training on education methods, control schools did not receive any intervention and neither intervention was available in the region [35]. The standard of care in Desai et al. was a short course of unsupervised iron supplementation which was given together with presumptive malaria treatment [37]. All four arms received a comparable standard of care involving one of the following interventions; daily-supervised iron supplementation, daily-unsupervised iron supplementation, twice weekly supervised, or twice weekly unsupervised iron supplementation. Philips-Howard et al. offered their intervention arm treated bed-nets but their control arm did not receive bed-nets during the study, as was the standard policy in the region in 1998/9 when the study recruited [38].

b. **Control arms with conditions worse than the stated local standard of care**

Two out of fourteen cluster trials offered the control arm less than the stated standard of care. Freeman et al. selected only schools that did not meet the government policy for latrines provision [28]. Schools in three intervention arms either received the full intervention consisting of hygiene promotion component, teacher training, and sanitation component (although the number of latrines was limited whatever the size of the school), or a partial intervention consisting of either the hygiene promotion component and teacher training, or only teacher training. One arm did not receive any component, which was less than the stated standard of care to provide a certain number of latrines, based on the size of the school, but was a reality for the selected schools and for many schools in the region.

Crump et al. offered either flocculant disinfectant or a commercially available product based on sodium hypochlorite for household based water treatment in either of two arms. Households in the third arm continued with traditional water treatment and storage practices involving fetching water from ponds and rivers, cloth filtration and decantation. The government had a policy to provide clean drinking through a scheme of various agencies, for which there would have been a standard set up charge as well as user fees, but less than half of rural households and two thirds of urban households had been reached [36]. Children in the control 'traditional’ treatment arm were not assisted in accessing this basic public service or any other alternatives available in the region.

c. **Intervention arms receiving conditions worse than the local standard of care**

Two out of fourteen studies evaluated an intervention that was arguably inferior to the government policy or local standard of care while their control arms received the standard of care. Kangwana et al. offered their intervention arm subsidized pediatric Artemether Lumenfantrine through retail outlets and requiring parents to pay a small amount for it, without professional diagnosis by a health worker, and sometimes being dispensed without a prescription or pharmacists’ advice [31]. Under the government’s stated standard of care, children attend a local healthcare facility and are assessed by a professional health worker who prescribes free Artemether Lumenfantrine if required.

In Suchdev et al., vitamin A and iron were ostensibly available in public health facilities but the trial offered micronutrient supplements with vitamin A and iron that cost two Kenyan shillings per sachet and were sold by community based vendors [27]. Children in the intervention arm may have received worse care than the government standard because they paid for micronutrient supplements although this cost may have been offset by cost of travel to public health facilities. However, children in the intervention arm did not receive attention from a health worker whereas children in the control arm received attention from a health worker and free supply of some micronutrients from public health facilities where available, although availability was inconsistent.

### Prior evidence of efficacy or effectiveness of test interventions

a. **Interventions with prior evidence of efficacy and some evidence of effectiveness**

Three out of fourteen cluster trials reported prior clear evidence of efficacy and some evidence of effectiveness for their interventions. Freeman et al. reported that previous randomized control trials showed water treatment, sanitation and hygiene promotion intervention in schools in low-income countries reduced pupils’ absence and a similar intervention in homes improved the health status of children under five [28]. It was not known how components of this intervention worked together or whether there was a gender specific effect.

Crump et al. reported several studies showing efficacy of flocculant disinfectant in developing countries, and one Kenyan study showed flocculant disinfectant was more effective than sodium hypochlorite in treating turbid water and for pathogens resistant to chlorine [36]. It was not clear how these effects would translate specifically to households. Philips-Howard et al. assessed the effectiveness of providing treated bed-nets to households and reported a previous randomized control trial in sub Saharan Africa showing treated bed-nets reduced all-cause mortality in children under five by seventeen percent, and a Tanzania program estimated a twenty-seven percent increase in survival in children under five, but had no information about the effect of treated bed-nets on mortality in very young children in areas with high malaria transmission pressure [38].

b. **Interventions with prior evidence of efficacy and no prior evidence of effectiveness**

Two of fourteen cluster trials reported clear evidence of efficacy but little or no evidence of effectiveness for their interventions. Suchdev et al. evaluated an intervention distributing micronutrient supplements using social marketing and had evidence micronutrient powders worked as well as prophylactics at reducing anemia, iron and vitamin A deficiency in young children [27]. Skarbinski et al. evaluated the effect of providing rapid diagnostic test kits for malaria in local health facilities, which was established to be faster and required fewer resources than traditional microscopy testing, but had not been previously used in Kenya and at the time were being introduced into the public healthcare system [34].

c. **Intervention with prior evidence of efficacy but mixed evidence on effectiveness**

Six out of fourteen cluster trials reported that interventions had clear evidence of efficacy as well as mixed evidence on effectiveness. Patel et al. investigated whether a school based handwashing, water treatment and provision, and hygiene promotion intervention improved hygiene practices and reduced illness and reported one systematic review and a meta-analysis showed handwashing with soap and household water treatment reduced illnesses and diarrhea but reported difficulties implementing handwashing and water treatment practices [26]. Three studies in developing countries showed similar interventions led to behavior change in hygiene practices in children and parents, and four school based Kenyan studies showed similar interventions led to reduction in school absenteeism. However, they reported a lack of evidence showing a direct impact on health.

Gewa et al. investigated whether an intervention providing school based food supplementation led to reallocation of household foods among household members [25]. Three studies in low income countries showed school based food supplementation programs increased children’s overall energy intake while one study showed household based food supplementation program led to redistribution of food to non-intended targets. Opondo et al. and Ayieko et al. assessed an intervention providing multifaceted implementation of pediatric guidelines in rural district hospitals on pediatric admission care [29,32]. These well established guidelines were developed by the World Health Organization for use by health facilities in restricted resources but few low-income countries had implemented or assessed these established guidelines, although it was theorized that multifaceted implementation in comparison to traditional implementation increased uptake of guidelines.

Kangwana et al. reported pilot programs providing subsidized Artemether Lumenfantrine in Uganda and Tanzania increased uptake with good adherence to subsidized prices and reduced the use of old malaria treatment regimens, while studies in West African testing similar programs showed inconsistent availability of subsidized Artemether Lumenfantrine and low adherence to recommended retail prices [31]. Desai et al. assessed the impact of an intervention providing supervised daily iron supplementation compared to other modes of supplementation to treat childhood anemia [37]. A 1999 meta-analysis showed supervision had an effect, and that daily dosing was more efficacious than weekly dosing in pregnant women, but did not show conclusive results in children or adolescents. Desai et al. reported that a study with aboriginal children published after the meta-analysis showed twice weekly supervised supplementation was better than unsupervised daily iron supplementation in reducing anemia.

d. **Interventions with prior partial or weak evidence of efficacy**

Three out of fourteen cluster trials reported their intervention had partial or existing but weak evidence of efficacy. Brooker et al. assessed whether an intervention combining school based intermittent malaria treatment and enhanced literacy instruction improved educational achievement [33]. A previous Sri Lankan study showed school based malaria treatment reduced anemia and improved exam scores, and a previous Kenyan cluster trial showed a similar intervention reduced anemia but had no effect on educational scores.

Clarke et al. evaluated whether school based malaria prevention led to improved educational achievement [35]. A previous study showed providing intermittent malaria prevention in schools led to lower rates of anemia and malaria, fewer clinical attacks and reduced malaria related absenteeism. Three African studies showed similar interventions reduced malaria prevalence and led to improved cognitive abilities. There was no evidence to link reduction in malaria related school absenteeism and malaria related anemia to improvements in cognition and educational performance.

Zurovac et al. investigated whether an intervention sending reminder text messages to health workers’ mobile phones improved their adherence to guidelines on malaria diagnosis and treatment for young children [30]. Two previous Kenyan randomized control trials evaluated text messages sent from health workers to HIV/AIDS patients to improve patients’ adherence to treatment regimens but there was no data on how this approach might improve adherence among professional health workers.

### Results of the trials

a. **Positive results**

Five of the fourteen cluster trials reviewed reported positive results for their interventions. Opondo et al. did a regression analysis of data collected by Ayieko et al. and found the odds ratio of children receiving inappropriate antibiotics for non-bloody diarrhea in control hospitals compared to intervention hospitals was 0.30 with 95% confidence interval (CI) 0.09 to 1.02) [29].

Zurovac et al. showed that sending text messages to health workers improved their adherence to malaria guidelines [30]. Effects for four treatment tasks and five out of six dispensing and counseling tasks showed an improvement of 21.4% with 95% CI 9.0 to 33.7, P-value = 0.0007 in the short term, and 23.7% with 95% CI 11.6 to 35.7, P = 0.0001 at six months. There was a smaller effect when all tasks were measured, 10.3%, 95% CI 4.0 to 16.6 P = 0.0013 in the short term and 11.3% at six months, 95% CI, 5.1 to 17.6, P = 0.0004.

Kangwana et al. found retail delivery of subsidized Artemether Lumenfantrine resulted in 35.9% of children receiving Artemether Lumenfantrine on the same day or next day in the intervention arm compared to 14% of children in control arms given 95% CI, P = 0.0002 and adjusted P = 0.0001 [31]. Tibamal, the intervention brand, accounted for 63% of Artemether Lumenfantrine sold. Ninety-three percent of Tibamal was bought at recommended price with standard deviation of 5.3% and there was no significant difference in adherence to dosages.

Desai et al. found daily iron supplementation was better than twice weekly supplementation in supervised clusters [37]. The mean difference at 6 weeks was 4.2 g/L and 4.4 g/L with 95% CI at twelve weeks. In unsupervised groups, no mean difference was noted at 6 weeks but at 12 weeks, a difference of 3.4 g/L P = 0.02 95% CI was noted between arms. Philips-Howard et al. found treated bed-nets provided a protective effect of 26% in children aged between 1 and 11 months and 14% efficacy in children aged 12 and 59 months for 95% CI [38].

b. **Mixed results**

Seven of fourteen cluster trials reported mixed findings. Gewa et al. found school based food supplementation did not significantly reduce quantity or quality of children’s intake at home but significantly reduced protein intake in parents in the vegetarian arm [25].

Patel et al. found hygiene promotion increased handwashing and reduced all illness by 5% versus 7%, with Estimated Difference in Median (EDM) 3%, 90% CI 4 to 1%, and reduced acute respiratory illness at first round by 2 versus 3% with EDM -2%, 90% CI -3 to -1%, and found similar rates of illness during the second round of measurements, but did not observe any effect on diarrheal diseases during both rounds [26].

Suchdev et al. found community based marketing of micronutrient supplements significantly reduced prevalence of iron deficiency by a difference of 14%, P = 0.001, noted a smaller reduction in vitamin A deficiency, difference of 5%, P = 0.01, and in anemia prevalence with 7.1% absolute difference, P = 0.1 [27].

Freeman et al. evaluated the impact of water treatment, hygiene promotion and sanitation in schools on attendance and by gender and found water treatment and hygiene promotion had no significant impact on overall absence even when sanitation was added [28]. However, their intervention had a gender specific effect: hygiene promotion and water treatment alone reduced the odds of absence in girls by 58%, odds ratio of 0.42, 0.21 to .085, but no effect on boys, odds ratio of 0.88, 0.45 to 1.71. The addition of sanitation showed comparable benefits for girls, odds ratio of 0.47, 0.21 to 1.05 but no effect for boys, odds ratio 0.98, 0.52 to 1.87.

The study by Ayieko et al. involved a multifaceted implementation of pediatric guidelines and observed general improvements across a total of 18 outcome measures including task/process and structure indicators to varying degrees [32]. For instance, completion of admission tasks was higher in intervention hospitals, uptake of recommended therapeutic practice was higher in intervention hospitals compared to intervention hospitals and the proportion of children receiving inappropriate doses of drugs was lower in intervention hospitals.

Clarke et al. provided intermittent preventative malaria treatment and an education intervention and found prevalence of malaria dropped in intervention schools to 6.3% and to 12.6% in the control schools, with adjusted risk ratio 0.52, 95% CI 0.29 to 0.93; P = 0.028, noted a mean increase in performance in two classroom based measures, in code transmission of 6.06%, 95% CI 2.83 to 9.27, P = 0.0007 and in counting test scores 1.80%, 0.19 to 3.41, P = 0.03, but found their intervention had no effect on educational achievement and on 2 other classroom scores [35].

Crump et al. found their intervention providing household based flocculant disinfectant significantly reduced diarrhea prevalence in children under 2, and flocculant disinfectant reduced prevalence by 25%, 95% CI -40 to -5, which was better than sodium hypochlorite reduction in prevalence by 17%, -34 to 4 compared to control practices [36]. In addition, flocculant disinfection significantly lowered turbidity to eight nephelometric units compared with 55 nephelometric units in other water treatment methods. There were significantly fewer deaths in households not using traditional methods, relative risk of death 0.58, P = 0.036, but the difference between either method was not significant, with 0.53 relative risk in flocculant disinfection, P = 0.052 compared to relative risk of 0.61, P = 0.108 in households using sodium hypochlorite.

c. **Negative results**

Two out of fourteen studies reported negative results. Brooker et al. reported intermittent malaria screening and treatment did not significantly affect anemia and malaria prevalence, classroom attention scores and educational achievement in older children, but noted an apparent negative effect on spelling scores in the younger class and on arithmetic scores [33].

Skarbinksi et al. found providing rapid diagnostic tests for malaria in health facilities increased their use from 35% to 46%, and reduced the use of traditional tests from 38% to 8%, but providing these kits did not change overall testing rate if measured by all methods [34]. However, provision of rapid diagnostic test kits reduced the use of clinical diagnosis of malaria to prescribe Artemether Lumenfantrine by 36%, P = 0.03. The study explained that these results were obtained because health workers in the study inappropriately substituted traditional tests for the rapid diagnostic test instead of using rapid diagnostic test kits in addition to other methods.

### Post-trial access to beneficial interventions

Five out of fourteen trials planned to offer their interventions, if successful, to their control clusters. The remaining nine studies did not report any arrangements for expanded access to control groups or rollouts. Only one of the five trials planning post-trial access was a pilot program and rolled out their intervention in the region. Suchdev et al. expanded provision of community based marketing of micronutrient supplements to include control villages after the trial (without promoting available free public services) [27].

The other four trials planning post-trial access were not pilot studies but still expanded access to control arms. Patel et al. rolled out their hygiene promotion and water and soap provision intervention to schools in the control arm. Brooker et al. planned, but did not implement intermittent malaria prevention and treatment and enhanced literary instruction in the control group, as the trial did not obtain positive results [33]. Freeman et al. provided latrines to schools in control arms after the trial [28]. Similarly, Philip-Howards et al. provided treated bed-nets to control arm families [38].

In two out of nine studies without prior arrangements for post-trial access to interventions, access for the wider population increased independent of results and of cluster trials. Kangwana et al. did not offer post trial access to subsidized Artemether Lumenfantrine but coverage rose to 60% during and soon after the trial as a result of parallel and independent government policy [31]. In Skarbinksi et al. the government independently increased the provision of rapid diagnostic tests from 25% to 50% according to plans during the trial as part of its own strategy, the authors explained negative results of the trial were caused by inappropriate use of tests during the study [34].

### Government involvement

Nine out of fourteen studies either used public facilities such as health facilities or schools, or involved public officials mainly health workers during design and implementation. Five out of fourteen studies reported developing their intervention with public sector partners. Zurovac *et al*. developed their mobile text message content with the Division of Malaria, Ministry of Health [30]. Kangwana *et al*. consulted with Pharmacy and Poisons Board who then gave permission for distribution of subsidized Artemether Lumenfantrine in retail outlets, implemented their trial through Division of Malaria, and collected data from the retail sector through Population Services International, a non-governmental organization [31]. In Ayieko *et al*. and Opondo *et al*., the Ministry of Health and pediatric teams assisted in multifaceted implementation of pediatric guidelines [29,32]. For Brooker *et al*., Division of malaria staff implemented intermittent malaria screening and treatment component in schools and schoolteachers implemented their literacy component [33].

Five out of the fourteen studies reported partnerships with non-governmental organizations. Gewa *et al*. partnered with Child Nutrition Program, Freeman *et al*. was embedded within a larger research project run by CARE, (Cooperative for Assistance and Relief Everywhere) a non-governmental organization [25]. Patel *et al*. and Suchdev *et al*. were embedded within a wider project, the Nyando Integrated Health Project, and Suchdev *et al*. implemented their study through the Safe Water and AIDS Project who also trained their community-based marketers [26,27]. Kangwana *et al*. partnered with Population Services International to implement their intervention [31].

## Discussion

Following the 2002 legal ruling in the Treatment Action Campaign case, the main legal question addressed in this review was whether the 14 cluster trials published since 2002 were warranted from the perspective of the child’s special human right to health which should guarantee access to treatment in the face of prior evidence supporting interventions that offered basic or life saving healthcare. State parties now have a clear negative duty not to limit access to proven and funded medicines by artificially restricting access to further 'pilot’ in certain areas, or by extension, run cluster trials.

As many as 11 of the 14 trials raised issues of human rights to health, and this is especially important as all had ethics approval from various ethics review committees, while 9 trials involved the use public facilities in recruitment or in implementation, or were facilitated by government bodies and thus were government sponsored or supported.

Four trials aimed to fill gaps in public provision of care or in public infrastructure, but instead developed interventions that fell below the regional standard of care. Two of these trials charged for malaria treatment or micronutrient supplements so as to increase coverage and distribution when government policy was to offer them free of charge to children who attended public health facilities and received attention from a health worker. The other 2 remaining cluster trials failed to provide control clusters with the standard expected of public services, one failing to assist families with very young children to access clean water despite a government policy to do so, and the other failing to provide sufficient numbers of latrines in control schools despite government standards for latrines in schools. This is an area for further development as it could be argued that interventions can offer lower than the stated standard of care if trials do not prevent participants from accessing the local standard of care and where the local stated standard of care is unmet. However, where the success and need for an intervention depends on unmet local standards, the right to health will require that such interventions, are closely developed in partnership with the relevant health authorities and that ethics review committees are cognisant of the need for justification for these interventions.

In three of eleven trials, there was a substantial amount of prior evidence of benefit (both of efficacy and of effectiveness), making it unclear why the trials were thought to be scientifically interesting or logistically necessary, and in one case, substantial risks encountered by one control arm were borne by very young children. These trials looked for increasingly specific sub-group effects such as gender or age, or sought to target the intervention to more specific groups, such as households, which could raise the issue of circular justification for trials, just in order to evaluate effects by such specific group or cluster.

Despite clear guidance and a publishing requirement under the CONSORT cluster statement in 2004, reporting of rationale for using cluster trial designs was generally inadequate across trials, this is problematic from a human right to health perspective, as a violation of children’s right to health by unnecessarily delaying full realization, and as it reduces visibility of cluster trials and children’s involvement and can thus lead to missed opportunities for protection of children.

Although two thirds of trials appeared to be designed to test effectiveness based on prior evidence of efficacy, only one third of reviewed cluster trials reported little prior evidence of efficacy, thus demonstrating a clear need for research. Retrospectively, however, only about half these trials fully corroborated prior evidence with positive results which may cast doubt on the value of prior evidence of efficacy. Ethics review committees have the onus of examining prior evidence of efficacy and effectiveness and it may be necessary to highlight the need to report and scrutinize prior evidence supporting cluster trials with children. It is still unclear exactly what prior evidence justifies restricting access through cluster trials, human rights law requires the progressive realization of, if not full respect for, children’s right to health, such that deviating from specific government policies to develop interventions lower than the stated or available standard of care or distribute health resources simply for the purposes of research (and sometimes regressing their realization in the process) raises concern. Where government policy states that a basic specific standard should be provided, yet its ambition is not yet fully adopted or realized, human rights law would require effort and resources to be better concentrated on furthering its ambition, especially in relation to essential services or basic healthcare for children.

In other respects, there seems to have been a steady improvement in design and implementation of cluster trials with children in Kenya since 2002 from a right to health perspective, as more recent trials offered active control conditions and a few recent trials planned to roll out successful interventions to control arms showing that from a local perspective, independent and government cluster trials recognize that control groups may have a claim to benefits. More recently, the Constitution of Kenya, 2010 clarified a minimum standard under children’s right to health which is basic healthcare, a key point for all stakeholders involved in cluster trials to note, and informing classic debates in global research ethics about trial conditions, and minimal or relative risk by appealing to standards local authorities *should* meet rather than what they provide in practice.

## Conclusion

A major challenge for children’s right to health in Kenya identified in this review was that most trials were not clearly justified since there was often prior evidence of efficacy and some evidence of effectiveness for their interventions. Some trials did not make provisions to implement results, and even evaluated interventions which did not seem to meet the local standard of care and minimum level of basic care required by children’s special right to health. In this regard, government should take greater heed of human rights perspectives, as they are often involved in design and implementation, as well as bearing ultimate responsibility for the ethics review of cluster trials. Government obligations are shared by stakeholders such as ethics review committees and host and facilitating institutions while other stakeholders such as international sponsors, international non-governmental organization and independent cluster trials also have obligations primarily towards children in control clusters as well as a wider obligation to maximize opportunities for providing access to successful results.

This review provides an essential outline of children’s right to health in cluster trials based on the South African legal precedent *Treatment Action Campaign and Others v Minister of Health and Others*, and on international and Kenya provisions for children’s right to health. The obligations discussed here under the children’s right to health are consistent with local ethical guidelines, and may address gaps in ethical guidelines and legal issues for cluster trials with children in sub Saharan Africa.

## Abbreviations

CARE: Cooperative for Assistance and Relief Everywhere; CI: Confidence interval; Cluster trials: Cluster randomized trials; CONSORT cluster statement: CONSORT statement, extension to cluster randomised trials; EDM: Estimated Difference in Median; HIV/AIDS: Human immunodeficiency virus/Acquired immunodeficiency syndrome; Kenya Guidelines: Guidelines for the Conduct of Biomedical Research involving Human Research Subjects in Kenya, National Council for Science and Technology Number 45, 2004; General Comment number 14: General comment 14. On the right to the highest attainable standard of health, Article 12 of the International Covenant on Economic Social and Cultural Rights. 2000/4; NGO: Non-governmental organization; Ottawa Statement: The Ottawa Statement on the ethical design and conduct of cluster randomized trials, 2012; RCT: randomized control trial; Treatment Action Campaign case: Treatment Action Campaign and Others v Minister of Health and Others.

## Competing interests

The authors declare that they have no competing interests.

## Authors’ contributions

EO: data collection and analysis, manuscript writing and final approval of manuscript. SE: primary conception, design, review and sampling of data, manuscript writing and final approval of manuscript. Both authors read and approved the final manuscript.
